# The Effect of Emulsifying Protein and Addition of Condensed Tannins on n-3 PUFA Enriched Emulsions for Functional Foods

**DOI:** 10.3390/foods9111589

**Published:** 2020-11-02

**Authors:** Susana Cofrades, Alba Garcimartín, Joaquín Gómez-Estaca, Francisco J. Sánchez-Muniz, Beatriz Herranz, Adrián Macho-González, Juana Benedí, María Dolores Álvarez

**Affiliations:** 1 Institute of Food Science, Technology and Nutrition (ICTAN-CSIC), 28040 Madrid, Spain; joaquin.gomez@csic.es (J.G.-E.); herranzh@vet.ucm.es (B.H.); 2 Pharmacognosy and Botany Department, Pharmacy School, Complutense University of Madrid, 28040 Madrid, Spain; a.garcimartin@ucm.es (A.G.); jbenedi@ucm.es (J.B.); 3 Nutrition and Food Science Department (Nutrition), Pharmacy School, Complutense University of Madrid, 28040 Madrid, Spain; frasan@ucm.es (F.J.S.-M.); amacho@ucm.es (A.M.-G.); 4 Department of Food Technology, Veterinary Faculty, Complutense University of Madrid, 28040 Madrid, Spain

**Keywords:** gelled emulsion, sodium caseinate, whey protein isolate, condensed tannins, rheology, lipid digestibility

## Abstract

This paper examines the effect of the type of the emulsifying protein (EP) (sodium caseinate (SC) and whey protein isolate (WPI)) on both oil-in-water liquid-like emulsions (Es) and the corresponding cold gelled emulsions (GEs), and also the effect of addition of carob extract rich in condensed tannins (T). The systems, intended as functional food ingredients, were studied in various different respects, including rheological behaviour, in vitro gastrointestinal digestion with determination of the release of non-extractable proanthocyanidins (NEPA) from T, antioxidant activity and lipolysis. EP significantly affects the rheological behaviour of both Es and GEs. T incorporation produced a structural reinforcement of GEs, especially in the case of SC. The digests from Es displayed a higher antioxidant activity than those from GEs. T lipase inhibition was observed only in the formulations with WPI. Our results highlight the importance, in the design of functional foods, of analyzing different variables when incorporating a bioactive compound into a food or emulsion in order to select the better combination for the desired objective, owing to the complex interplay of the various components.

## 1. Introduction

In recent years, the use of structured liquid oils from different origins in the form of emulsion gels has been proposed as a novel technological approach to replace saturated fats in order to improve the quality of different foods (meat products, spread products, bakery products, etc.). In this sense, oils are structured to create a plastic fat, which retains solid-like properties while possessing a healthier fatty acid profile [1].

Solid-like emulsions are generated from stable liquid-like oil-in-water emulsions (Es) by gelling the continuous phase and/or aggregating the emulsion droplets. Generally, the gelation process involves thermal, enzymatic or chemical treatments, but recently the use of cold gelling agents as polysaccharides, proteins and their combination with other ingredients has been proposed. They are able to form a continuous network through polymer interactions, which are responsible for the functional properties of structured emulsion gels [2,3]. These gelled emulsions (GEs) can also act as a vehicle for several bioactive compounds that are different from healthy fatty acids. It was also reported that properties of GEs depend on the nature and complex interactions among their structural components [4].

In this regard, Pintado et al. [3] determined the technological and structural characteristics of GEs formulated with olive oil and chia flour and seeds as emulsifiers, and a cold gelling agent (microbial transglutaminase (MTG), alginate or gelatin), obtaining different relationships between structural properties and textural behavior. These same authors underlined the importance of understanding the complex interactions among the different components of GEs through textural and structural methods to facilitate their incorporation into a suitable food matrix to replace fats in healthy products. Therefore, an improved understanding of the role of each GE component and their interaction would help to develop strategies to modify or design new healthy products. In addition, the composition and structure of the GE systems may play an important role in oil digestion activity by modifying lipid hydrolysis. In this sense, recent studies have suggested that lipid digestion may be potentially influenced by different factors: (a) the nature of the emulsifier/stabilizer used to form emulsions, which affects interfacial phenomenon, (b) oil droplet size, which has an effect on the surface area available for lipase adsorption, and (c) consistency of the systems, which affects mass transport [5,6].

Thus, it would be interesting to design GEs elaborated with different emulsifying proteins (EPs) and a cold gelling system (a mixture of gelatin, *κ-*carrageenan and MTG) together with a healthy lipid combination (olive, linseed and fish oils), which is designed with polyunsaturated fatty acids (PUFAs) in suitable proportions for purposes of achieving recommended intake objectives [7]. Natural biopolymer molecules (proteins and polysaccharides) can prevent droplet aggregation and stabilize emulsions by forming a thin layer adsorbed on the oil-in-water surface, therefore reducing interfacial tension [8]. Thus, they are extensively used as functional ingredients in the food industry to stabilize emulsions and foams, control the texture and structure of foods, and protect and deliver bioactive ingredients to targeted sites [9]. Proteins are complex macromolecules with different levels of structure that form an immobile viscoelastic adsorbed layer on the surfaces of oil droplets, which mechanically prevent coalescence [5]. Among the proteins that are most widely used as emulsifiers of food-related emulsions are sodium caseinate (SC) and whey protein isolate (WPI) from milk, due to their excellent functional and nutritional properties [4]. However, to the best of our knowledge, there is scarce information in the scientific literature about their role in structured emulsions, including their implication on digestion. In this context, it would be interesting to evaluate the effect of these proteins on the structure of Es and GEs, and their potential relationship with in vitro lipid digestion.

PUFAs are prone to lipid oxidation. Among other strategies, the addition of water-soluble pro-oxidants or antioxidants has been effective in limiting oxidation processes [10]. Carob fruit extracts contain condensed tannins (T) in the form of non-extractable proanthocyanidin (NEPA), primarily known for either their antioxidant activity [11] or hypolipemic properties [12]. Furthermore, polyphenols (both extractable and NEPA) have health benefits when consumed thanks to their capacity to decrease oxidative stress, and they have been shown to ameliorate some chronic diseases in which insulin resistance is produced [13]. Therefore, the incorporation of carob extract in the systems would have the double function of preventing lipid oxidation and inhibiting pancreatic lipase activity, thus promoting health when consumed.

The objective of the present work was to get a deeper insight into the structure–functionality relationship of n-3 PUFA-enriched Es or GEs as fat substitutes depending on the emulsifying protein (EP), and the addition of T with known antioxidant and hypolipemic properties.

## 2. Materials and Methods

### 2.1. Materials and Reagents

Extra virgin olive oil (Carbonell Virgen Extra; SOS Cuétara SA, Madrid, Spain), linseed oil (Natursoy S.L.; Alimentos Ecológicos, Castellterçol, Spain) and fish oil (Omevital 18/12 TG Gold; Cognis GmbH, Illertissen, Germany) were used as lipid phases in emulsion preparations. SC (Excellion EM 6; *Friesland Campina* DMV, Veghel, the Netherlands) and WPI (Provon 295; Glanbia Nutritionals, Kilkenny, Ireland) were used as emulsifying proteins (EPs). Condensed tannins (T) extracted from carob fruit were obtained from Biosearch S.A. (Granada, Spain). Bovine gelatin (200–220 bloom) was obtained from Manuel Riesgo, S.A. (Madrid, Spain), *κ*-carrageenan from Trades S.A. (Barcelona, Spain) and MTG from Ajinomoto (Tokyo, Japan). All other reagents were of analytical grade and acquired from Panreac Quimica, S.A. (Barcelona, Spain).

### 2.2. Methods

#### 2.2.1. Formulation and Preparation of Es and GEs

The composition of the emulsions and their process of elaboration were optimized in previous tests to obtain appropriate physicochemical characteristics [14]. Firstly, Es were prepared by mixing four different aqueous phases with SC or WPI (added at optimal emulsification amounts of 2 and 6 g/100 g, respectively) in the presence or not of T (3.9 g/100 g emulsion), and with a lipid phase consisting in a mixture of olive oil, linseed oil and fish oil (44.4, 37.9 and 17.7 g/100 g, respectively), in accordance with Delgado-Pando et al. [7]. Coarse emulsions were elaborated by the dropwise addition of the lipid phase (50 g/100 g) into a homogenizer (Thermomix™ 31, Vorwerk España M.S.L., S.C, Madrid, Spain) containing the different aqueous phases (50 g/100 g) while mixing at 3250 rpm for 20 min at room temperature. The coarse emulsions were passed once through a high-pressure homogenizer (GEA Niro Soavi MODEL Panda Plus 2000, Parma, Italy) at 55/7 MPa (first-stage pressure/second-stage pressure) to obtain fine emulsions. The same procedure was used to prepare the emulsions with T, which were added after each emulsion preparation and subsequently mixed. Es prepared with SC or WPI are referred to as E-SC-C and E-WPI-C, respectively, and used as controls, whereas those containing SC or WPI with added T are referred to as E-SC-T and E-WPI-T.

To structure or gel the different Es, *κ*-carrageenan (0.3 g/100 g emulsion) and bovine gelatin (0.5 g/100 g emulsion) were dissolved in deionized water (20 mL/100 g emulsion) at 80 °C and then added into 100 g of the emulsions, which were stirred at 1625 rpm/37 °C in the Thermomix, to avoid a possible thermal shock. Then, MTG (1.5 g/100 g emulsion) was dispersed in deionized water (10 mL/100 g emulsion) and added to the mixture. Aliquots of 80 mL were rapidly filled into 100 mL containers and thereafter stored at 4 °C for 24 h to obtain two GEs with SC or WPI (GE-SC-C and GE-WPI-C), both used as controls, and another two containing T (GE-SC-T and GE-WPI-T). Emulsions were stored in refrigeration (4 °C ± 2) until they were used.

#### 2.2.2. Droplet Size Distribution of Es

After diluting between 8- and 10-fold with distilled water, the particle size and distribution of oil droplets in the Es were determined with a Malvern Mastersizer S laser diffraction particle size analyzer (Malvern Instrument Ltd., Worcestershire, UK) equipped with a He-Ne laser (*λ* = 633 nm). The refractive index of the disperse phase was 1.53 and the refractive index of the dispersion liquid (distilled water) was 1.33. The measurement range was 0.05–900 μm. Obscuration was in the range of 8–15%. Particle size calculations were based on the Mie Scattering theory. Particle size measurements are reported as the volume-weighted mean or volumetric diameter, *d*_43_ (μm). Measurements were done at least in triplicate.

#### 2.2.3. Rheological Measurements

The rheological properties of both Es and GEs were carried out with a Kinexus pro rotational rheometer (Malvern Instruments Ltd., Worcestershire, UK), equipped with a cone and plate geometry (4° cone angle, 40-mm diameter) for measuring Es, and with a parallel-plate geometry (20-mm diameter and 1-mm gap) for GEs. Before measurement, both Es and GEs were tempered at ambient temperature. GEs were cut from graduated plastic tubes into disk-shaped slices, 20-mm in diameter and 1-mm thick, with a stainless steel cell specially designed for this diameter [15]. All samples were allowed to rest for 15 min before analyses to ensure both thermal and mechanical equilibrium at the time of measurement. Samples were covered with a thin film of Vaseline oil (PRS-Codex, Barcelona, Spain) to avoid evaporation. The temperature was controlled to within 0.1 °C by a Peltier element in the lower plate kept at 25 °C, except when non-isothermal heating processes were carried out. All measurements were done at least in triplicate.

##### Steady Shear Rheological Measurements of Es

The steady shear measurements were performed for shear rates ranging from 0.1 to 100 s^−1^. Data from the flow curves were fitted to the power law model ($\sigma =K{\dot{\gamma }}^{n}$), where *σ* (Pa) is the shear stress, *K* (Pa s^n^) is the consistency index and *n* is the flow index. Viscosities at 10 and 100 s^−1^ (*η*_10_ and *η*_100_) were also derived from flow curves and compared to *K* values from power law fits, which correspond to the apparent viscosity at a shear rate of 1 s^−1^ (*η*_1_).

##### Dynamic Rheological Measurements of GEs

To determine the linear viscoelastic (LVE) region, stress sweep tests were run at 1 Hz with the shear stress (*σ*) of the input signal varying from 0.02 to 200 Pa. Frequency sweep tests were run to obtain the mechanical spectra of GEs. Samples were subjected to strain that varied harmonically with time at variable frequencies from 0.01 to 10 Hz. The strain amplitude was set at *γ* = 0.5% within the LVE range. The complex modulus (G*), storage modulus (*G*′), loss modulus (*G*″), and loss factor, tan *δ*, were determined as functions of frequency. Creep and recovery tests were also conducted on GEs. For this purpose, an instantaneous stress *σ*_0_ (ranging between 1.91 and 35.1 Pa, depending upon formulation), corresponding to 0.5% shear strain within the LVE range, was applied for 600 s in the creep tests and the resulting change in strain over time *γ*(*t*) was monitored. When the stress was released, recovery was also recorded for 600 s. The creep and recovery results were described in terms of the shear compliance function, *J*(*t*) = *γ*(*t*)/*σ*_0_. From *J*(*t*) data, the relaxation modulus *G*(*t*) was obtained and used to find the gel strength (*S*) and relaxation exponent (*n*) [16]. Finally, temperature sweep tests were performed for dynamic thermomechanical analysis from 20 to 80 °C at a linear heating rate (2 °C min^−1^). Frequency (1 Hz) and strain *γ* = 0.5% (within the LVE range) were fixed.

#### 2.2.4. Simulated In Vitro Gastrointestinal Digestion of Es and GEs

The digestion process in a human gastrointestinal tract was simulated through an in vitro static digestion model, which was a modified version of that described by Laparra et al. [17]. Ten g of Es or chopped GEs were accurately weighed and transferred to Erlenmeyer flasks in triplicate. Then, 90 mL of distilled water were added and the mixture was acidified to pH 2 with 0.1 M HCl. For the gastric step, pepsin (0.2 mg/mL) was added, the volume was adjusted to 100 mL, and the flasks were placed in a thermostatic bath at 37 °C for 2 h with continuous shaking. The gastric step was stopped by placing the flasks in an ice bath and then the pH was adjusted to 6.5 with 1 M NaHCO_3_. The intestinal phase of digestion was then initiated by the addition of a pancreatin (50 µg/mL)−bile solution (300 µg/mL) and incubation in a thermostatic bath at 37 °C for 2 h with continuous shaking; subsequently, the reaction was stopped by again placing the flasks in an ice bath. The final pH of the solution was adjusted to 7.2 with 0.5 M NaOH. For NEPA from T and antioxidant activity determinations, the remainder-undigested gel was removed by filtering through mesh of 0.16 mm in diameter, and the filtrate concentrated by freeze-drying. Simulated in vitro gastrointestinal digestion protocols commonly separate the soluble fraction by filtering through Whatman no. 1 filter paper or centrifuging, in order to evaluate accessibility. By filtering the digest through the 0.16 mm mesh, released T are also being considered, and they are expected to play a relevant beneficial role after colonic fermentation [18]. For the analysis of lipid hydrolysis, the digested whole sample was used for lipid extraction, which was performed as described below.

##### Released Non-Extractable Proanthocyanidins (NEPA) from T and Antioxidant Activity

NEPA extraction was carried out from the freeze-dried digested according to the procedure described by Pérez-Jiménez et al. [19]. Ten mL of butanol/HCl (97.5:2.5, *v*/*v*) containing 0.7 g of FeCl_3_ were added to this residue and heated at 100 °C for 60 min. Then, samples were cooled to room temperature and centrifuged at 509× *g* for 15 min and the supernatant was collected. The residue was re-extracted with 5 mL of the above solution twice and all extracts were combined to obtain the total amount of 25 mL. The absorbance was measured at 555 and 450 nm in a Shimadzu UV-1800 spectrophotometer (Shimadzu Inc., Kyoto, Japan). Standard curves were obtained for carob pod tannin concentrate. The results were expressed as mg of NEPA released/100 g emulsion.

An automated photochemiluminescent system (Photochem^©^, Analytik Jena Model AG; Analytic Jena USA, The Woodlands, TX, USA), which measures the capacity to quench superoxide free radicals, was used to determine the antioxidant activity in the NEPA extracts. In brief, 20 μL of the extract containing NEPA were added to reagent kits provided by the manufacturer and the system measured the total antioxidant capacity. Trolox was used as a standard, the samples were measured in triplicate and results were expressed in Trolox Equivalents (mmol TE/g sample).

##### Extent of Lipolysis during In Vitro Digestion

Once in vitro digestion was finished, fat was extracted following the method described by Cofrades et al. [20]. Briefly, the resulting digest was mixed twice with chloroform/methanol (1:1, *v*/*v*) (Sigma-Aldrich, Madrid, Spain). The organic phase was collected and then purified using a chloroform/methanol/0.9% NaCl solution mix (vol:vol, 3/48/47). Finally, the obtained solution was dehydrated by filtering it through anhydrous sodium sulfate (Merck, Madrid, Spain), evaporated in rotavapor at 40 °C, and subjected to nitrogen stream for 3 min until the isolated lipid phase was obtained. High Performance Size Exclusion Chromatography (HPSEC) was performed to elucidate the triglycerides (TGC), diglycerides (DGC), monoglycerides (MGC) and free fatty acids (FFA) composition and contents, in both the non-digested and digested samples, as described by González-Muñoz et al. [21]. Fifteen mg of extracted fat diluted in 1 mL of tetrahydrofuran were applied in HPSEC (Agilent 1100 series, Madrid, Spain) with a 20 μL sample loop. A refractive index detector (Agilent Technologies 1260 infinity, Madrid, Spain) and two serially-connected 300 mm × 7.5 mm i.d. (5 μm particle size), 0.01 and 0.05 μm, PL gel columns (Agilent, Bellefonte, PA, USA) were operated at 40 °C. HPLC grade tetrahydrofuran was used as the mobile phase with a flow of 1 mL/min. TGC, DGC, MGC and FFA were quantified as g/100 g of sample. The specific refractive index increment (dn/dc value) observed between the sample and the solvent was used for the different compounds’ quantitation after accepting equal chromatographic response for all triglyceride-derived compounds. The content of the different groups of compounds, expressed as percentage on oil, were calculated as follows: 100*(A/∑) in which A is the area of the corresponding peak and ∑ is the sum of areas for all peaks. In order to verify the peak assignation, standard retention times of TGC, DGC, MGC, and FFA were obtained after injecting an iso-molecular mix of tripalmitin, triestearin, triolein and trilinolein in the case of TGC; dipalmitin, distearin, diolein, dilinolein in the case of DGC; monopalmitin, monostearin, monolein and monolinolein in the case of MGC; and palmitic, stearic, oleic and linoleic acids for FFA. The three samples from each group were measured in duplicate.

### 2.3. Data Analysis

For rheological and droplet size measurements, two-way analysis of variance (ANOVA) was performed to study the main effects separately: emulsifying protein (EP), presence of condensed tannins (T) and the interaction effect (EP × T). As a significant interaction effect was observed in most of the rheological parameters evaluated, one-way ANOVA was performed, comparing the means of the same T level (without and with) for the two EPs and of the same EP for both presence and absence of T. In addition, for lipid composition, NEPA and antioxidant activity after in vitro digestion, the effect of cold gelling systems (E versus GE) was also studied by using the unpaired Student’s *t*-test. Minimum significant differences were calculated using Fisher’s least significant difference (LSD) test at 5 and 1% levels. Analyses were done using IBM SPSS Statistics for Windows, version 25.0 (IBM Corp., Armonk, NY, USA).

## 3. Results and Discussion

### 3.1. Droplet Size Distribution of Es

**Figure 1:**
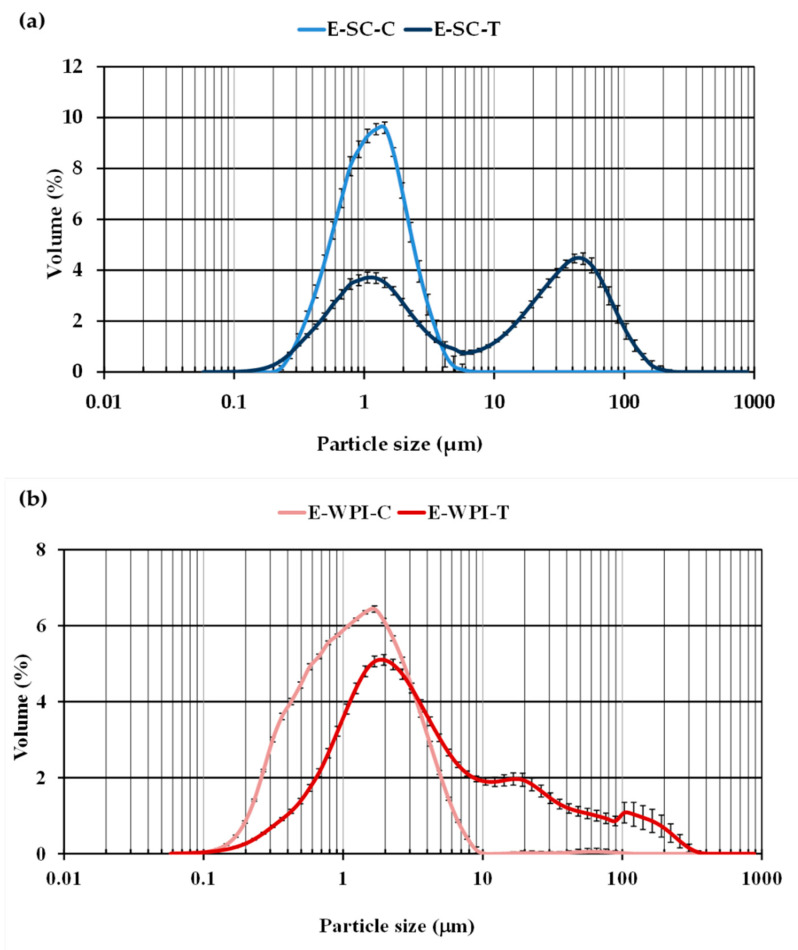
Droplet size distributions for liquid-like emulsions (Es): (**a**) stabilized by SC without and with added condensed tannins (E-SC-C and E-SC-T); (**b**) stabilized by WPI without and with added condensed tannins (E-WPI-C and E-WPI-T).

Figure 1 shows the droplet size distribution of E-SC-C and E-SC-T (Figure 1a) and of E-WPI-C and E-WPI-T (Figure 1b). As may be observed in Figure 1, both control Es exhibited a monomodal distribution with a mainly large-sized population (around 1.4 μm), which was narrower and better defined in the case of E-SC-C, likely reflecting a more stable emulsion. Similar results were observed by Jiang et al. [22] in caseinate-based emulsions elaborated with increasing low molecular weight emulsifier concentrations, but exhibiting a bimodal size distribution at a concentration below 0.25% *w/w*. These findings indicate that in control Es, both EPs, SC and WPI, were adequately adsorbed onto the droplets’ surface, forming an interfacial membrane through repulsive strong interactions (e.g., steric and electrostatic interactions) between oil droplets, which are resistant to rupture [8,23].

On the other hand, the incorporation of T produced larger droplets with an increase in the width of distribution, thus significantly changing the profile of the droplet size distribution in the case of both EPs. More specifically, E-SC-T (Figure 1a) showed a bimodal pattern with two main similar populations of droplets with sizes of around 1 and 45 μm, respectively. Both distribution peaks could be related to individual oil droplets and aggregates of oil droplets, respectively. In turn, E-WPI-T exhibited one larger-sized population around 1.4 μm, as also observed for E-WPI-C (Figure 1b), and two minority populations with peaks at around 20 and above 100 μm, respectively. Hence, the presence of T might interfere between the oil droplets and the protein as a filler, therefore decreasing the interfacial tension and producing in some way self-aggregation of protein molecules, causing coalescence and/or flocculation with larger-size droplets. According to Trujillo-Cayado et al., [24] the occurrence of a second population of oil droplets could be the consequence of recoalescence phenomena during the high-pressure homogenization process.

Particle size measurements *d*_43_ were also obtained for all the four Es. The *d*_43_ values were significantly lower for E-SC-C (1.23 ± 0.084 µm) than for E-WPI-C (1.70 ± 0.285 µm). The faster the emulsifier adsorption, the smaller the droplets of the emulsion formed [22]. The *d*_43_ value increased very significantly with additional T incorporation in Es prepared with both EPs (23.8 ± 0.829 and 16.6 ± 2.87 µm, respectively). However, the contrary was observed in the control Es, where *d*_43_ was significantly higher in the E-SC-T sample than in the E-WPI-T one. A previous study showed that phenolic compounds mainly interact with casein rather than with whey protein [25], which is reflected by a much higher aggregate size of casein globules implying strong hydrophobic bindings of T with caseinates. In Es stabilized by SC and WPI, Chang and McClements [6] reported lower and similar *d*_4,3_ values between them (0.283 ± 0.003 and 0.225 ± 0.006 µm, respectively) than those found in this study, although differences could be ascribed to the different formulation and conditions of preparation of the Es made by these authors.

As a result, SC and WPI seem to be good emulsifiers, and therefore they could form stable and homogeneous emulsions, although it is likely that they show a different rate of lipid digestibility [6]. Furthermore, the presence of T yielded higher mean diameters and different droplet size distributions, indicating that T incorporation could lead to the occurrence of coagulation or coalescence phenomena, mainly in the case of Es prepared with SC, due to the higher affinity of polyphenols for this milk protein. In addition, these structural changes produced by T presence in the formulations could have consequences on expected antioxidant and hypolipemic activities.

### 3.2. Rheological Properties of Es and GEs

#### 3.2.1. Steady Shear Rheological Properties of Es

**Figure 2:**
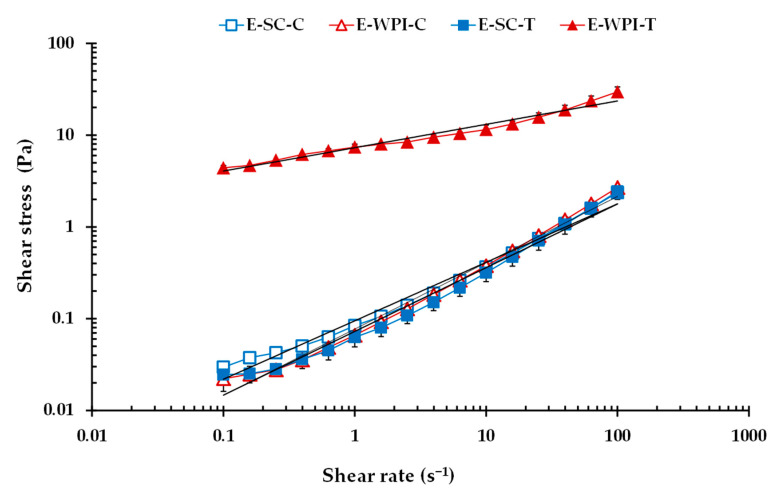
Typical flow curves and applicability of the power law model to liquid-like emulsions (Es) stabilized by two emulsifying proteins (SC and WPI), without (E-SC-C and E-WPI-C) and with added condensed tannins (T) (E-SC-T and E-WPI-T).

The flow behavior of an emulsion is critical to its physical stability [22]. Figure 2 illustrates the applicability of the power law model to the Es stabilized by both SC and WPI, either without or with added T. Shear stress–shear rate plots become almost linear when plotted on double logarithmic coordinates, and the power law model properly described the data of Es, which exhibited a shear-thinning behavior. However, the emulsion E-WPI-T became more viscous, showing the highest resistance to flow and the highest shear stress value over the complete shear rate range studied, and even with an apparent and measurable initial yield stress at~3 Pa, indicating plastic behavior. E-WPI-T also had apparent viscosity values similar to those reported by Jiang et al. [22] in caseinate-based Es stabilized with low molecular weight emulsifiers. On the contrary, small differences can be observed between the flow curves of E-SC-C, E-SC-T and E-WPI-C samples, mainly at the highest shear rates.

The parameters *K* and *n* are determined from the plots of log *σ* versus log $\dot{\gamma }$, where each resulting straight line’s intercept is log *K* and the slope is *n*. Table 1 shows all the steady shear parameter values of Es. Based on the *R*^2^ determination coefficient values, E-WPI-T exhibited the worst power law fit, probably due to the fact that the power law model disregards yield stress. With regard to the effect of the EP used in the formulation, in both Es without and with added T, there were significant differences (*p* < 0.05) between the *K*, *n*, *η*_10_ and *η*_100_ values of the two emulsions, although the effect was much more significant in emulsions with added T. Therefore, structural differences of Es formulated with different EP are greatly magnified when the bioactive compound is added, which shows the importance of the type of EP in the final characteristics of the system, which may ultimately condition a greater or lower effect of the bioactive compound.

E-SC-C showed significantly higher *K* and lower *n* values than E-WPI-C, although it also showed significantly lower *η*_10_ and *η*_100_ values. However, E-WPI-T sample presented the highest *K*, *η*_10_ and *η*_100_ values, and E-SC-T the lowest ones. In addition, all the Es showed pseudoplastic behavior (*n* < 1), but mainly E-WPI-T brought about a significant lowering of the flow index (*n*), that is to say, the values were further removed from Newtonian behavior (*n* = 1), indicating higher pseudoplasticity and greater resistance to the flow. In accordance with Pal [26], an increase in droplet size is accompanied by a decrease in the degree of shear thinning behavior in concentrated emulsions, as observed in this study for E-SC-T as compared to E-WPI-T (Table 1). In contrast, other authors observed that with increasing cluster size, the viscosity increases due to an increase in effective oil volume fraction [27].

According to the T effect, in samples prepared with WPI, the incorporation of T significantly increased the values of *K*, *η*_10_ and *η*_100_, and significantly decreased the *n* value (Table 1). On the contrary, the incorporation of T in samples prepared with SC only had a significant effect on the *n* value, and both E-SC-C and E-SC-T had weaker, looser structures than their WPI counterparts. Therefore, in Es, the effect of the presence of T was dependent on the EP used to stabilize the emulsion. The much lower viscosity of E-SC-T compared to E-WPI-T could be attributed to the fact that with SC and T, there were fewer interactive emulsion droplets due to increased particle size.

A liquid-like protein-stabilized emulsion is characterized by its oil volume content, its droplet size distribution and the protein surface coverage, which forms an immobile viscoelastic adsorbed layer, at the oil–water interface [4]. Significant differences in the behavior of E-SC-C and E-WPI-C emulsions must be related to the protein system used to stabilize the Es, as well as to droplet size distribution, because the lipid material was the same in all cases. Both SC and WPI emulsifiers are soluble/dispersible mixed milk protein ingredients, although SC is a mixture of flexible proteins, whereas WPI is a mixture of globular ones [6].

On the other hand, the T used in our experiments contain 34–48% NEPA and 0.5–1% soluble extractable polyphenols [12], and a high affinity between polyphenolic compounds of crude extracts and casein has been previously observed [25]. Therefore, it is likely that the T that interacted with the SC protein remained in the protein matrix. On the contrary, the authors just cited observed very little interaction between WPI and pure phenolic compounds. Additional functional ingredients may also lead to long-term transient gelation by the depletion flocculation mechanism [28], which could justify the existence of a yield stress in the E-WPI-T sample, and in its resultant weak gel-like structure. A solid-like emulsion gel may be generated from a stable liquid-like emulsion by gelling the continuous phase and/or aggregating the emulsion droplets, and aggregated droplets may be present if there is an excess of unadsorbed protein (depletion flocculation) [29].

#### 3.2.2. Dynamic Rheological Properties of GEs

##### Frequency Sweep Tests

**Figure 3:**
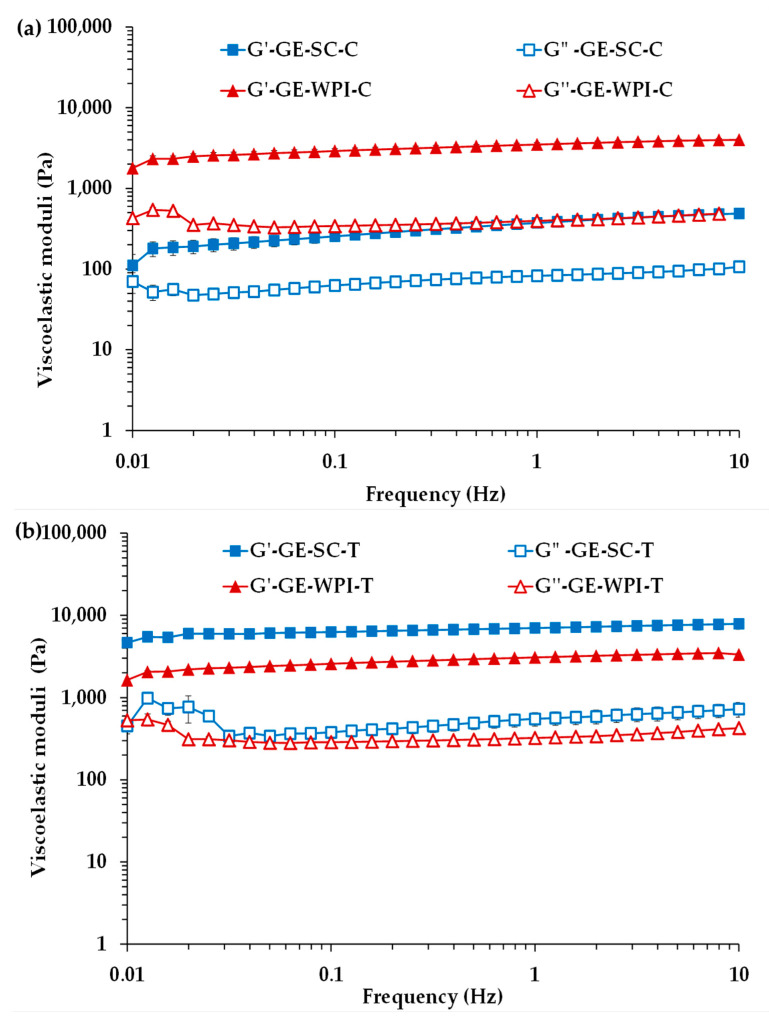
*G*′ and *G*″ as functions of frequency for gelled emulsions (GEs): (**a**) stabilized by SC and WPI without added condensed tannins (GE-SC-C and E-WPI-C); (**b**) stabilized by SC and WPI with added condensed tannins (GE-SC-T and GE-WPI-T).

It is useful to determine dynamic rheological properties of GEs to study the contribution of the EP to emulsion stability and the effect of droplet–droplet interactions on the viscoelasticity of the system [30]. Mechanical spectra of GE-SC-C, GE-WPI-C, GE-SC-T and GE-WPI-T are shown in Figure 3. All the GEs exhibited solid-like elastic-dominant behavior because storage modulus (*G*′) was higher than loss modulus (*G*″) over the entire frequency range. This result indicates that the cold gelling system used, a mixture of gelatin, *κ-*carrageenan and MTG, produced the formation of interactive and viscoelastic gel systems in all the Es. It is well known that MTG forms GEs with enhanced rheological properties and stability by covalent cross-linking by acyl transfer between glutamine and lysine residues in proteins [3,14,31]. Therefore, GEs can be classified as weak physical gels with predominantly solid-like character [32].

Moreover, in all GEs, *G*′ was practically frequency-independent, whereas *G*″ exhibited a tendency to increase at lower frequencies (from 0.1 to 0.01 Hz). This increase was even more remarkable in the presence of T (Figure 3b). The relative *G*″ increases observed between 0.1 and 0.01 Hz could be attributed, at least partially, to a possible denaturation of the proteins in the structured systems because of longer durations of the shear. Then, they may unfold their secondary and tertiary structure, allowing the absorption of water molecules, so they could swell, and an increase in the *G*″ values, therefore causing the three-dimensional network to lose its stability. This can be explained by a partial disruption of the adsorbed protein layer that stabilized the network, which was more significant in the GEs with added T. A greater accessibility of glutamine and lysine residues by MTG due to structure unfolding could also contribute to the observed increase in *G*″.

The effects of EP and T incorporation on dynamic rheological properties at 1 Hz are shown in Table 2. GE-WPI-C showed significantly higher *G*′ and *G*″ values and lower tan *δ* ones than GE-SC-C. This last result is in accordance with the fact that the gap between *G*′ and *G*″ was greater in the GE-WPI-C sample than in the GE-SC-T one (Figure 3a). Therefore, GE-WPI-C, having a higher arginine content than its SC counterpart [14], exhibited a more strongly connected structure and higher viscoelasticity. Molecular weights of monomeric forms of casein proteins (15–26 kDa) are quite similar to those of the major proteins from WPI (18–66 kDa) [33,34]. However, when compared with whey proteins, caseins are particularly disordered and considerably hydrophobic, which facilitates their rapid adsorption during emulsification and leads to a rapid formation of a thick sterically stabilizing layer that protects against flocculation and coalescence [34], resulting in much lower viscoelasticity.

On the other hand, when T were incorporated in the emulsions, both structured GEs had viscoelastic moduli values that were much higher than in their respective control counterparts (Table 2). In addition, this increase was much more significant when SC was used as emulsifier. Therefore, it seems that T addition caused a structural reinforcement, being especially notable in GE-SC-T, as it was also evidenced by its significantly lower tan *δ* value reflecting higher viscoelasticity and more elastic gel-like behavior. Note that tan *δ* values, which indicate the amount of energy lost to the amount of energy stored, were lower than 1 but close to 0.1; this result also reflects a weak gel behavior. On the other hand, interactions between T and SC were stronger than between T and WPI, as reported previously [14,25]. In similar cold-set GEs, these authors reported that casein proteins, rich in proline and other apolar amino acids and with a more open random structure, are more prone to interact with T than with WPI.

In addition, GEs can also be characterized using the concept of weak gel [35]. According to this weak gel model, weakly structured food systems can be characterized by a three-dimensional network where rheological units are bound by weak interactions [36]. In this study, rheological units would represent cross-links between milk protein molecules, and between T and proteins in GEs with added T, assuming that the contribution to the rheological properties of the cold gelling system was the same in all systems. The dynamic data for these systems are described by a power law Equation (1) relating dynamic complex modulus (G*) and frequency (*f*):

G*(*f*) = *Af*^1/z^ (1)

where *z* is the “coordination number”, which is the number of flow units interacting with one another within the three-dimensional network, and it can be assumed as an extent of interactions [36], and *A* is a constant that can be interpreted as the “interaction strength”. It is worth noting that the *A* parameter is equal to the complex modulus (G*) evaluated at a frequency (*f*) of 1 Hz.

Values of *A* and *z* obtained from fits of G* values to power laws between 10 and 0.1 Hz are also shown in Table 2. Data between 0.1 and 0.01 Hz were discarded because the observed *G*″ increase in this frequency decade would significantly affect the linearity of G* versus frequency. Strength and development of GEs networks were influenced by both the EP and T effects studied. As it could be expected from the elastic gel-like behavior observed, the *A* and *G*′ values of all the GEs were very similar, and with the same significant differences between them (Table 2). Therefore, the strength of the interactions was also significantly weaker (*p* < 0.05) in control GEs than in those with added T. This result reflects that T increase the effectiveness of protein adsorption, decreasing protein content in the continuous phase, and making the network more resistant to permanent deformation. In addition, the effect was mainly significant in GE-SC-T, also evidenced by a greater extent of interactions (higher *z* value) reflecting a higher stability of GE-SC-T, as indicated above. This reinforcement caused by the interaction between T and SC could increase the thickness of the protein viscoelastic film around the fat droplets, influencing lipid digestibility as described below. There were no significant differences between the *z* values of WPI GEs with and without added T, meaning a similar number of interactions and degree of organization (Table 2). Nevertheless, the significantly lower developed strength of interactions of GE-WPI-T as compared to GE-SC-T could imply that in the system GE-WPI-T, T were located far from the lipid phase [14].

Han et al. [25] also reported that casein is more prone to interact with polyphenols than whey proteins. On the other hand, conformational changes such as structural unfolding of caseins could allow greater reactivity of MTG with glutamine and lysine [33] increasing viscoelasticity and improving the conformational stability of this gelled emulsion.

##### Creep and Recovery Tests

Creep-recovery analysis is a transient test performed at constant stress within the LVE range. It produces creep and recovery compliances *J*(*t*) over longer-time scales than oscillatory rheological measurements [37], and consequently these experiments can cause irreversible breakdown of short-range interactions. Hence, they can provide additional information of the contribution of the type of EP and the addition of T on *J*(*t*) and, therefore, on the network stability over longer-time scales than oscillatory tests.

**Figure 4:**
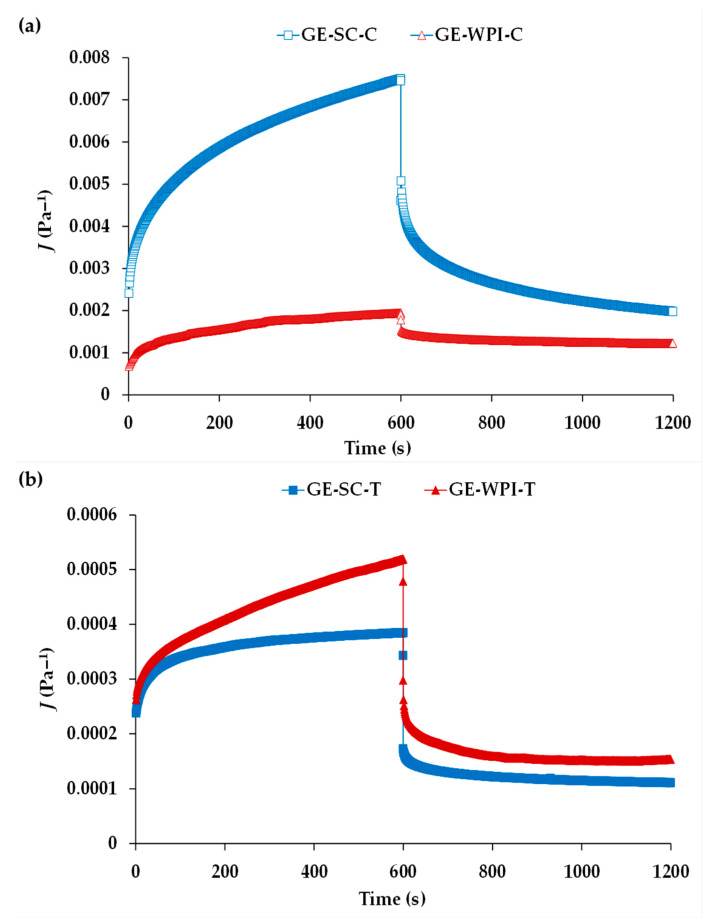
Evolution of creep and recovery compliance *J*(*t*) values for gelled emulsions (GEs): (**a**) stabilized by SC and WPI without added condensed tannins (GE-SC-C and E-WPI-C); (**b**) stabilized by SC and WPI with added condensed tannins (GE-SC-T and GE-WPI-T).

Figure 4a,b show the mean creep-recovery compliances *J*(*t*) for GEs without and with added T, respectively. GE-SC-C (Figure 4a) showed much higher values of compliance *J*(*t*) than GE-WPI-C during both the creep and recovery stages. This indicates that during loading, there was more sequential rupturing of cross-links (probably weak interactions) in GE-SC-C than in the GE-WPI-C gelled system. In addition, the GE-WPI-C sample showed a more complete recovery (Figure 4a), indicating that it was a more elastic network, as it is also evidenced by its lower tan *δ* values (Table 2).

The addition of T (Figure 4b) changed this rheological behavior pattern completely. First, both GEs showed much lower *J*(*t*) values than those of their control counterparts, corroborating the structural reinforcement produced by T incorporation, especially in GE-SC-T showing lower *J*(*t*) values than GE-WPI-T. These data corroborate those observed from the frequency sweeps. However, note that samples with added T also showed less recovery upon load removal (Figure 4b). This fact would indicate that for longer loading times, GEs with added T could suffer more structural collapse and irreversible breakage of interactions, which is associated with poorer binding properties [14].

Data from Figure 4 make it possible to derive the relaxation modulus *G*(*t*) [37], which provides the “gel strength” (*S*) and “relaxation exponent” (*n*) by means of Equation (2):

*G*(*t*) = *S*.*t*^−*n*^ (2)

As indicated previously, T produce stronger GEs, as also shown by their significantly higher *S* values as compared to their control counterparts (Table 2), making these gelled systems more resistant to deformation, especially in the case of using SC as EP. This suggests that both EPs (SC and WPI) formed an adsorbed layer around the oil droplets, likely of different thickness, because the proteins unfold and rearrange their different secondary and tertiary structures to expose hydrophobic residues to the hydrophobic phase [23], which allows interaction with its neighboring protein molecules to form this adsorbed layer. Hence, the stability of the GEs depends on the different structure of the adsorbed protein. T could modify the structure of the adsorbed protein or act as a filler between this layer and the droplets, because of the affinity that polyphenols show towards food proteins [25]. This is very remarkable in the case of SC, so that the presence of T completely changes the structural configuration from a flexible and weak network to the most rigid and stable protein network, as shown by their higher *S* value and lower *n* one (Table 2). The lower the *n* value, the higher the density of physical cross-linking reactions, which increases the extension of the junction zones and the degree of connectivity, also indicating a better organized and cohesive network [38]. Therefore, T improved the structural stability in the presence of SC more significantly, which may be explained by a stronger interaction of this protein with polyphenols than that of WPI [25]. The results confirm the effects of T on the strength of interactions between rheological units.

In summary, the above results reflect the stronger and more elastic network with a high degree of connectivity of GE-SC-T ensuring that the nature of interactions in the protein network is preserved.

##### Temperature Sweep Tests

**Figure 5:**
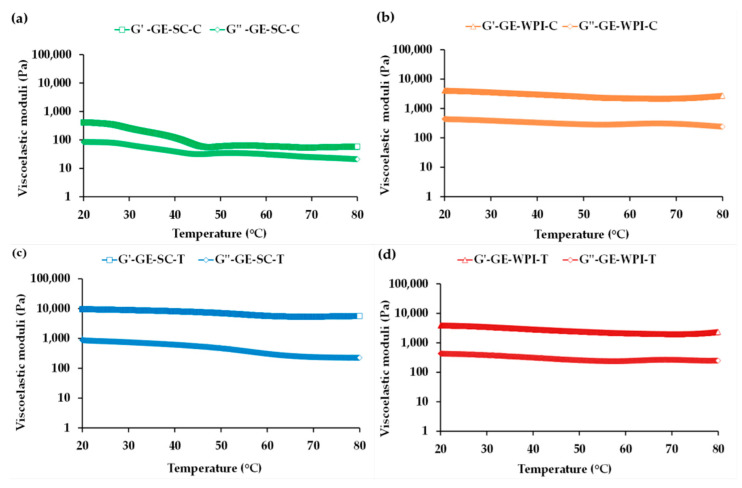
Temperature sweeps of *G*′ and *G*″ for gelled emulsions (GEs): (**a**) stabilized by SC without added condensed tannins (GE-SC-C); (**b**) stabilized by WPI without added condensed tannins (GE-WPI-C); (**c**) stabilized by SC with added condensed tannins (GE-SC-T); (**d**) stabilized by WPI with added condensed tannins (GE-WPI-T).

Figure 5 shows the influence of temperature within the LVE range in terms of storage (*G*′) and loss moduli (*G*″) to analyze the temperature stability of GEs. As these GEs are intended to be used as functional fat replacers in different food matrices suffering thermal treatment during their processing, their thermo-rheological behavior is crucial in terms of their applicability. The thermal profile of GE-SC-C (Figure 5a) indicated a gel weakening with increasing temperature, as reflected by a decrease in both *G*′ and *G*″ from 20 up to 80 °C. Nevertheless, GE-WPI-C (Figure 5b) showed a much more stable profile, keeping *G*′ one order of magnitude greater than *G*″ over the whole temperature range.

In turn, in the case of GEs with added T, GE-SC-T (Figure 5c) showed a more pronounced decrease in *G*″ than GE-WPI-T (Figure 5d), but in both structured systems temperature hardly affected gel elasticity. It is worth noting that the WPI profiles are very similar in the absence and presence of T.

This result evidences that, with the exception of GE-SC-C, in all other GEs there is only a slight loss of structural configuration by the rupture of weak intermolecular bonds between the adsorbed proteins as temperature increases. Therefore, mainly GE-SC-T, but also GE-WPI-C and GE-WPI-T, may be suitable as fat replacers in foods, including meat products, subject to high temperatures.

### 3.3. Simulated In Vitro Gastrointestinal Digestion of Es and GEs

#### 3.3.1. Released Non-Extractable Proanthocyanidins (NEPA) from T and Antioxidant Activity

The amount of NEPA released (accessible to be fermented by microbiota and accordingly absorbed) at the end of in vitro gastrointestinal digestion, as well as the corresponding antioxidant activity, are shown in Table 3. As expected, the four systems without added T (E-SC-C, E-WPI-C, GE-SC-C and GE-WPI-C) showed no NEPA content. However, they did present some antioxidant activity, which is mainly attributed to proteins and peptides released during digestion processes, although the contribution of antioxidants from the oils cannot be discarded. Samples with added T (E-SC-T, E-WPI-T, GE-SC-T and GE-WPI-T) showed a significantly higher amount of NEPA and antioxidant activity (*p* < 0.05) than their respective controls, and thus it can be deduced that NEPA was the main cause of the antioxidant activity of either Es or GEs, although T interactions with other components could affect the accessibility of this ingredient, and therefore its antioxidant properties [11].

With regard to the effect of emulsion structuration by cold gelation, the digests from Es exhibited a significantly (*p* < 0.05) higher NEPA content and much higher antioxidant activity than their gelled counterparts GEs. Therefore, NEPA were more effectively retained within the gelled matrices, which impaired their release. This is largely attributed to an incomplete release of T from GEs during digestion, as some debris was observed in these samples when the digests were filtered.

Discussing the effect of EP on control systems, only GE-WPI-C had significantly (*p* < 0.05) higher antioxidant activity than its GE-SC-C counterpart. On the contrary, regarding T-systems, the two systems elaborated with SC (E-SC-T and GE-SC-T) had a significantly higher amount of NEPA and antioxidant activity than their counterparts formulated with WPI (Table 3), suggesting a different behaviour of samples when T were incorporated to the system, which is in line with the rheological results. Therefore, as can be seen in the table, 100 g of E or GE will provide ≈ 450–250 or ≈ 350–150 mg NEPA, respectively, depending on the EP (SC or WPI), which would reach the colon in free form, exerting their antioxidant capacities and being able to be fermented by microbiota. It is well known that NEPA exert an important biological action in the colon, improving antioxidant and antiproliferative capacities, reducing intestinal tumorigenesis and modifying gene expression, as has been observed in different animal models [19]. Moreover, NEPA are extensively fermented by the action of microbiota, giving place to absorbable metabolites, which are also involved in interesting systemic effects [18].

Finally, in all the systems, the presence of T improved (*p* < 0.05) their antioxidant activities very significantly (Table 3), as was expected, corroborating previous findings [11]. Therefore, T incorporation is a convenient strategy for enhancing antioxidant capacity and retarding lipid oxidation in these systems.

#### 3.3.2. Extent of Lipolysis in Es and GEs

Table 4 summarizes lipid composition after in vitro digestion of the four liquid-like emulsions (Es), and the four gelled emulsions (GEs), with and without T. The extent of lipolysis was affected by the EP used, by the formulated system, and finally by T incorporation. Hence, the three factors must be taken into account.

The effects of the delivery system (Es or GE) and EP (SC or WPI) are discussed together since they are interconnected. The comparison of lipid profile from formulated systems (Es and GEs) revealed higher fat digestion in GEs, especially in the case of those containing WPI, showing less triglycerides (TGC) or more free fatty acids (FFA) than their Es counterparts, in both C and T samples. In a previous paper [20], we demonstrated that gelatin and MTG do not have any impact on pancreatic lipase activity; hence, the promotion on lipolysis by GEs should be attributed to their structural characteristics. Regarding the effect of EP, SC and WPI showed significant differences in lipolysis but with opposite trends depending on the delivery system used. Thus, Es containing SC (with and without T) showed a greater extent of lipolysis than those with WPI, as can be observed in the significantly lower levels of diglycerides (DGC) observed compared with E-WPI-C (−16.8%, *p* < 0.01), along with higher levels of monoglycerides (MGC) and FFA (21.6% and 13.6%, respectively; *p* < 0.01). Such differences would be partially explained because of the smaller droplet size (lower *d*_4,3_) found in E-SC as compared with E-WPI, which would have allowed a better access of pancreatic lipase to the emulsified lipid in the surface of SC-coated droplets than in that of WPI-coated droplets. Similarly, the initial SC layer surrounding the droplets did not prevent the formation of FFA in corn oil-in-water emulsions [39]. In addition, the results obtained by Borreani et al. [40] also showed that lipase was able to access the emulsified lipid more readily in a conventional emulsion with calcium caseinate-coated droplets than in other cellulose ether-coated droplets. Similar results were found in emulsions made with SC as emulsifier, which showed a greater lipid digestion than the whole oil [20]. On the contrary, regarding GEs, GE-SC-C was more resistant to digestion, with a significantly higher TGC level (23.9%, *p* < 0.001) and lower DGC, MGC and FFA levels (74.6%, 50% and 21.9%, respectively) than GE-WPI-C. A suitable explanation of this result is that offered by Pang et al. [41], who showed that gelatin can significantly modify GE properties as a function of pH and temperature, showing a greater loss of structure in those emulsions made with WPI. Taking these data into account, it is possible that the higher lipid digestion found in GE-WPI-C is due to a greater system rupture in the in vitro gastric stage. Furthermore, it can be observed that when gelatin is not used (E-WPI-C), fat digestion is lower compared with GE-WPI-C and also compared with E-SC-C, confirming the pH gelatin-weakness hypothesis. The described rheological properties differ with lipid digestion results; as it has been pointed out, GE-WPI-C samples showed lower values of compliance and a more complete recovery, with a high MTG-gel-strengthening effect, along with more elastic behavior compared to GE-SC-C (Figure 4a). However, following the conclusions of Pang et al. [41], the cause can be related to gastric rheological changes due to pH-sensitivity, which has not been studied this time. Furthermore, it must be remembered that the in vitro digestion protocol chosen could be considered as an initial phase of the intestinal stage. Thus, a gastric rupture of GE-WPI-C would promote a faster fat digestion at this early point than GE-SC-C would.

Apart from the type of EP and the formulated systems, T addition is another important factor to consider. The results obtained clearly indicate a different effect of T on lipolysis depending on the emulsifying protein (EP) used. In this sense, in the systems containing SC, T addition involved the promotion of fat digestion, and did not display the expected pancreatic lipase inhibition [12,13]. As can be seen in Table 4, E-SC-T showed a significantly lower amount of DGC and a higher amount of MGC compared with the E-SC-C sample, while GE-SC-T presented significantly lower TGC and higher MGC levels than GE-SC-C. The lack of the expected hypolipemic effect of T, when incorporated to SC-T systems, could be connected with T-SC interactions. The necessary interaction of T and lipase to cause its inhibition may be sterically hindered if T remain bound to SC. On the other hand, the higher fat digestion found in SC-T systems compared to SC-control ones, which should be linked to the important structural changes caused by T addition, as the rheological results demonstrated, remains to be explained. As shown above, the incorporation of T to E-SC decreased viscosity with respect to E-SC-C, facilitating enzyme digestion access. Regarding GE-SC-T results, with the greatest lipolysis among SC-systems, they can be due to the rheological behavior pattern. In connection with the previous discussion (Figure 4b), GE-SC-T showed less recovery, suggesting more risk of structural collapse and irreversible breakage of interactions, which can be responsible for the observed lipid profile. Finally, digestion results concur with NEPA released in SC-T systems, as they show the highest amount of NEPA in free form, indicating an easier digestion.

In contrast to SC, both systems elaborated with WPI and T (E-WPI-T and GE-WPI-T) displayed lipid digestion reduction, as compared to those without T. This delay in lipid digestion may be the result of two causes: (a) structural changes due to the addition of T; and (b) a direct T inhibitory effect on pancreatic lipase. Both E-WPI-T and GE-WPI-T systems exhibited a significantly higher amount of TGC, and lower amount of DGC, as compared with their counterparts without added T (Table 4), although the effect of T was more pronounced in the GE system. The results suggest different behavior patterns regarding structural changes. Firstly, E-WPI-T presented pseudoplastic properties with higher viscosity and resistance than E-WPI-C. For its part, the GE-WPI-T sample presented high deformability (Figure 4b), evidencing a greater rupture of cross-links that could facilitate droplets to become flocculated or coalesced in the gastrointestinal tract. Then, the surface area of lipids exposed to proteolytic enzymes is reduced, thus impairing fat release and slowing down lipid digestion. Concerning lipase inhibition, it is important to point out that NEPA release in WPI-samples was lower than in SC-samples (Table 3). However, in WPI-samples, when NEPA were released, it appears that they were capable of interacting with and blocking lipase. This would not happen in SC samples due to the interaction between SC-T, as detailed above. In this sense, the lack of the expected hypolipemic effect of T, when incorporated into SC-T systems, could be connected to T-SC interactions. The necessary interaction of T and lipase to cause its inhibition may be sterically hindered if T remain bound to SC.

Lastly, T-systems have been compared among each other. E-SC-T showed significantly higher lipid digestion than E-WPI-T, while both GE-T systems (GE-SC-T and GE-WPI-T) displayed a similar degree of lipid digestion, counterbalancing the strong difference between their control counterparts.

## 4. Conclusions

The results obtained make it possible to conclude that the emulsifying protein and/or addition of the bioactive compound (T) significantly modify the structure of the systems, the release of the bioactive compound, the antioxidant activity and lipid digestion. Formulating the system using SC as the emulsifying protein cancels the inhibiting effect of T on pancreatic lipase activity completely. Due to their structural/rheological characteristics, SC-T systems present greater lipid digestion compared with the controls, which implies an opposite effect from that expected. Thus, it is of great importance to verify that bioactive compounds maintain their potential effect once added to a system or particular matrix in order to formulate a functional food. The election of the system to be used will be adapted to the objective pursued. Accordingly, if the purpose is to develop a system able to release a healthy type of fat (e.g., rich in n-3 PUFAs), the system to be chosen would be GE-WPI-C, since it allows greater lipid digestion. If T were added in search of an antioxidant effect, it should be considered that fat would be digested to a lesser extent due to the lower bioavailability of the GE-WPI-T system. On the other hand, E-SC-T would be the most adequate option if the goal is to diminish the absorption of a certain type of fat (e.g., rich in SFAs) and to increase the antioxidant activity in the digestive tube of the consumer.

## Figures and Tables

**Table 1:** Effect of emulsifying protein (EP) and condensed tannins (T) on the steady shear rheological properties of liquid-like emulsions (Es).

Liquid-like Emulsions (Es)	*K* (Pas*^n^*)	*n* (-)	*R* ^2^	*η*_10_ (Pa s)	*η*_100_ (Pa s)
E-SC-C	0.095 ± 0.001 ^Aa^	0.637 ± 0.010 ^Bb^	0.984 ± 0.002	0.036 ± 0.001 ^Ba^	0.024 ± 0.001 ^Ba^
E-WPI-C	0.077 ± 0.001 ^Bb^	0.723 ± 0.001 ^Aa^	0.988 ± 0.004	0.038 ± 0.001 ^Ab^	0.027 ± 0.000 ^Ab^
E-SC-T	0.073 ± 0.015 ^Ba^	0.696 ± 0.017 ^Aa^	0.979 ± 0.005	0.031 ± 0.004 ^Ba^	0.024 ± 0.003 ^Ba^
E-WPI-T	7.22 ± 0.272 ^Aa^	0.250 ± 0.019 ^Bb^	0.957 ± 0.009	0.849 ± 0.0.073 ^Aa^	0.296 ± 0.039 ^Aa^

Values are given as mean (*n* = 3) ± standard deviation. A, B: effect of EP. For each rheological property and the same T level (without or with), mean values without the same letter are significantly different (*p* < 0.05). a,b: effect of T incorporation. For each rheological property and the same EP, mean values without the same letter are significantly different (*p* < 0.05). *K* and *n*: consistency and flow behavior indexes from the power law model, respectively; *η*_10_ and *η*_100_: viscosities at 10 and 100 s^−1^ from flow behavior curves; (-): dimensionless.

**Table 2:** Effect of emulsifying protein (EP) and condensed tannins (T) on dynamic rheological properties (at 1 Hz), weak gel model parameters and creep rheological properties of gelled emulsions (GEs).

Gelled Emulsions	*G*′ (Pa)	*G*″ (Pa)	tan *δ* (-)	*A* (Pa s^1/z^)	z (-)	*R* ^2^	*S* (kPas*^n^*)	*n*	*R* ^2^
GE-SC-C	373 ± 36.5 ^Bb^	82.4 ± 6.93 ^Bb^	0.221 ± 0.003 ^Aa^	375 ± 38.3 ^Bb^	7.09 ± 0.669 ^Bb^	0.990 ± 0.009	0.514 ± 0.122 ^Bb^	0.222 ± 0.022 ^Aa^	0.993 ± 0.004
GE-WPI-C	2485 ± 53.0 ^Ab^	278 ± 4.15 ^Ab^	0.114 ± 0.001 ^Ba^	2508 ± 54.5 ^Ab^	13.1 ± 0.603 ^Aa^	0.998 ± 0.001	1.79 ± 0.372 ^Ab^	0.185 ± 0.034 ^Aa^	0.984 ± 0.018
GE-SC-T	6990 ± 937 ^Aa^	552 ± 102 ^Aa^	0.079 ± 0.004 ^Bb^	7025 ± 102 ^Aa^	19.8 ± 0.231 ^Aa^	1.000 ± 0.000	5.57 ± 1.85 ^Aa^	0.083 ± 0.013 ^Bb^	0.982 ± 0.014
GE-WPI-T	3813 ± 209 ^Ba^	411 ± 23.4 ^Aa^	0.109 ± 0.001 ^Aa^	3840 ± 209 ^Ba^	14.1 ± 0.233 ^Ba^	0.999 ± 0.000	5.01 ± 0.507 ^Aa^	0.141 ± 0.034 ^Aa^	0.996 ± 0.002

Values are given as mean (*n* = 3) ± standard deviation. A, B: effect of EP. For each rheological property and the same T level (without or with), mean values without the same letter are significantly different (*p* < 0.05). a, b: effect of T incorporation. For each rheological property and the same EP, mean values without the same letter are significantly different (*p* < 0.05). *G*′, storage modulus; *G*″, loss modulus; tan *δ*, loss factor (= *G*″/*G*′). *A*, “interaction strength”; *z*, “coordination number”; *S*, gel strength; *n*, relaxation exponent; *R*^2^, determination coefficient; (-): dimensionless.

**Table 3:** Non-extractable proanthocyanidins (NEPA) released (mg/100 g) and antioxidant activity (mg Equation Trolox/100 g) after simulated gastrointestinal in vitro digestion of the different emulsions developed.

Added T		E-SC	E-WPI	GE-SC	GE-WPI
mg NEPA released/100 g E or GE	C	-	-	-	-
	T	432 ± 44 ^Ax^	344 ± 18 ^Bx^	235 ± 12 ^Ay^	167 ± 8 ^By^
mg Equation Trolox released/100 g E or GE	C	152 ± 11 ^Axb^	148 ± 39 ^Axb^	104 ± 6 ^Byb^	140 ± 5 ^Axb^
	T	1396 ± 162 ^Axa^	1060 ± 110 ^Bxa^	580 ± 28 ^Aya^	485 ± 36 ^Bya^

Values are given as mean (*n* = 3) ± standard deviation. Different letters in the same row (A, B) indicate significant differences (*p* < 0.05) as a function of EP (SC, WPI) for each emulsion structuration level (E, GE). Different letters in the same row (x, y) indicate significant differences (*p* < 0.05) as a function of cold gelation (E, GE) for each EP (SC, WPI). Different letters in the same column (a, b) indicate significant differences (*p* < 0.05) as a function of T (C, T) for each system studied.

**Table 4:** Lipid composition after in vitro digestion of liquid-like and gelled emulsions (Es and GEs). as a function of emulsifying protein (EP), condensed tannins (T) incorporation and cold gelation.

Lipid Composition	Added T	E-SC	E-WPI	GE-SC	GE-WPI
TGC	C	63.6 ± 1.32	64.4 ± 2.56 ^xb^	63.0 ± 1.22 ^Aa^	48.0 ± 5.90 ^Byb^
	T	64.1 ± 1.59 ^Bx^	68.5 ± 1.96 ^Axa^	59.6 ± 1.57 ^yb^	55.4 ± 2.37 ^ya^
DGC	C	13.4 ± 0.50 ^Axa^	16.1 ± 1.41 ^Ba^	10.2 ± 0.91 ^Ay^	17.8 ± 1.40 ^Ba^
	T	10.7 ± 0.04 ^Bb^	13.6 ± 1.57 ^Ab^	11.0 ± 0.59 ^A^	12.2 ± 0.69 ^Bb^
MGC	C	4.77 ± 0.52 ^Ayb^	3.74 ± 0.20 ^By^	5.72 ± 0.13 ^Axb^	8.58 ± 1.54 ^Bx^
	T	6.28 ± 0.74 ^Aa^	3.39 ± 0.31 ^By^	6.73 ± 0.32 ^Aa^	8.26 ± 0.49 ^Bx^
FFA	C	18.3 ± 1.08 ^Ay^	15.8 ± 1.28 ^By^	21.1 ± 0.29 ^Ax^	25.7 ± 3.54 ^Bx^
	T	18.9 ± 0.89 ^A^	14.5 ± 1.40 ^By^	22.7 ± 2.53	24.2 ± 1.68 ^x^

Values are given as mean (*n* = 3) ± standard deviation. Different letters in the same row (A, B) indicate significant differences (*p* < 0.05) as a function of EP (SC, WPI) for each emulsion structuration or cold gelation level (E, GE). Different letters in the same row (x, y) indicate significant differences (*p* < 0.05) as a function of cold gelation level (E, GE) for each EP (SC, WPI). Different letters in the same column (a, b) indicate significant differences (*p* < 0.05) as a function of T (C, T) for each system studied. TGC: triglycerides; DGC: diglycerides; MGC: monoglycerides; FFA: free fatty acids.
